# Transplantation of mesenchymal stem cells for spinal cord injury: a systematic review and network meta-analysis

**DOI:** 10.1186/s12967-021-02843-0

**Published:** 2021-04-28

**Authors:** Wei-can Chen, Wei-feng Liu, Yu-yan Bai, Ying-ying Zhou, Yan Zhang, Cong-mei Wang, Shu Lin, He-fan He

**Affiliations:** 1 Department of Anesthesiology, The Second Affiliated Hospital of Fujian Medical University, Quanzhou, China; 2 Centre of Neurological and Metabolic Research, The Second Affiliated Hospital of Fujian Medical University, Quanzhou, China; 3 Diabetes and Metabolism Division, Garvan Institute of Medical Research, Sydney, NSW Australia

## Abstract

Spinal cord injury (SCI) is a severe traumatic disease of the central nervous system, with a global prevalence of 236–4187 per million people. This meta-analysis aimed to evaluate the safety and efficacy of mesenchymal stem cells (MSCs) in treating patients with SCI as well as the optimal source and transplantation method of MSCs. PubMed, OVID, Cochrane, Web of Science, and China Biomedical Database were searched up until April 01, 2021. The study was conducted for five endpoints: American Spinal Injury Association (ASIA) motor and sensory score, ASIA grade improvement, Barthel Index (BI), and adverse reactions. Standard meta-analysis and network meta-analysis were performed using Stata 14.0. Eighteen studies with a total of 949 patients, were included in the meta-analysis. Standard meta-analysis showed that MSCs significantly improved ASIA motor score (P < 0.001), sensory score (P < 0.001), ASIA grade (P < 0.001), and BI (P < 0.001) compared to rehabilitation. In addition, in the network meta-analysis, autologous MSCs significantly improved the ASIA motor [MD = 8.01, 95% CI (4.27, 11.76)], sensory score [MD = 17.98, 95% CI (10.04, 25.91)], and BI [MD = 7.69, 95% CI (2.10, 13.29)] compared to rehabilitation. Similarly, compared to rehabilitation, intrathecal injection (IT) of MSCs significantly improved the ASIA motor [MD = 7.97, 95% CI (4.40, 11.53)] and sensory score [MD = 19.60, 95% CI (9.74, 29.46)]. Compared to rehabilitation, however, only the IL of MSCs was associated with more adverse reactions [OR = 17.82, 95% CI (2.48, 128.22)]. According to the results of SUCRA, both autologous MSCs and IT transplantation approaches most improved the neurological function in SCI patients. Cell transplantation using MSCs is effective in patients with SCI and IT of autologous MSCs may be more beneficial.

**Supplementary Information:**

The online version contains supplementary material available at 10.1186/s12967-021-02843-0.

## Introduction

Spinal cord injury (SCI) has a high disability rate and often leads to paraplegia or quadriplegia, urinary incontinence, and sensory dysfunction. The prevalence of spinal cord injury worldwide is 236–4187 per million people [1], with as many as 770,000 new cases per year [2]. In addition to personal suffering, socio-economic costs are also high due to the loss of labor force for many SCI patients and the increased assistance required by the caregivers and families [3]. However, substantial medical costs do not improve patient prognosis. The prognosis of patients with SCI remains abysmal, the mortality rate is still high, and life expectancy is significantly shortened [4]. Hence, there is an imperious necessity to develop an effective treatment strategy to treat patients with SCI.

Numerous basic and clinical researches have confirmed that secondary injuries, including local vascular damage and ischemia, oxidative stress, excitotoxicity, and inflammation/immune response, are the leading causes of further SCI damage [5]. The inflammatory response plays a vital role in both the acute and chronic stages of SCI. The initial reaction to SCI is by the resident glial cells, which recruits neutrophils, monocytes, and macrophages, followed by a gradual infiltration of B lymphocytes, T lymphocytes, and antigen-presenting cells [6]. These cells mediate the inflammation development, depending on the background, the duration of injury, and release several inflammatory factors, chemokines, second messengers, and reactive oxygen species leading to an imbalance in the local inflammatory microenvironment in SCI [7]. Moreover, it also aggravates the spinal cord tissue damage by further inflammation, including demyelination, damage repair, and scar hypertrophy [8]. Currently, a variety of treatment strategies for SCI have been developed, including drug therapy, surgery, and rehabilitation, but their therapeutic effect is not significant.

Cell transplantation therapy is a promising therapeutic strategy to replace the damaged nerve cells and/or create an environment conducive to repair. Various cell types, such as glial cells, neural progenitor/stem cells, and mesenchymal stem cells (MSCs), are candidates for SCI transplantation treatment [9]. Among them, MSCs promote neuronal survival and regeneration through the synthesis of neurotrophic and angiogenic factors, and have high biosafety and immunomodulatory properties, making them the most promising cell type for stimulating nerve regeneration [10]. The efficacy and safety of MSC transplantation have been demonstrated in several animal models of SCI [10]. A meta-analysis showed that MSCs transplantation could improve sensory function in patients with SCI; however, its effect on motor function is unclear [11]. Overall, the safety and effectiveness of MSCs application in patients with SCI remains controversial, especially for the selection of autologous and allogeneic MSCs and the cell transplantation methods.

Therefore, we performed a standard network meta-analysis of the most recent evidence to evaluate the efficacy and safety, as well as explore the optimal cell sources and approaches of MSC transplantation in the treatment of SCI.

## Materials and methods

This systematic review and meta-analysis followed the guidelines of the Preferred Reporting Items for Systematic Reviews and Meta-Analyses (PRISMA) [12].

### Search strategies

Qualified studies were systematically searched for in the PUBMED, OVID, China Biomedical Database (CBM), Web of Science, and Cochrane databases (all dates through April 01, 2021). The references of related reviews and meta-analyses were searched manually. The literature search strategy consisted of MeSH terms and the free words, "spinal cord injury" and "mesenchymal stem cells" (Additional file 1: Table S1).

Two researchers (Wei-can Chen and Yu-yan Bai) independently formulated the search strategy, conducted a pre-examination, checked, and determined the search strategies before conducting a formal search. In case of any dispute, both parties reach a consensus through discussion and, if required, referred to a third party (Shu Lin) for decision.

### Study selection criteria

#### Inclusion criteria

(a) Patients diagnosed with SCI, regardless of race, sex, age, disease course, and severity, were included in this study. (b) The treatment group was treated with autologous or allogeneic MSCs. Patients in the control group received rehabilitation treatment but did not receive stem cell therapy. (c) Had at least one of the following outcome indicators: (1) sensory and motor function measures: the American Spinal Injury Association (ASIA) score; (2) Living ability assessment scale: Barthel Index (BI); (3) and anticipated or had unexpected adverse reactions and mortality. (d) The type of study was randomized controlled trials or other controlled studies.

#### Exclusion criteria

(a) Studies that were on non-human subjects. (b) The data in the study being relevant but could not be extracted. (c) Studies that were not controlled studies, such as case reports, reviews, meetings, letters, surveys, or satisfaction studies. (d) Inclusion criteria were not met.

### Data extraction and quality assessment

Two researchers (Wei-can Chen and Wei-feng Liu) independently extracted the data and cross-checked it. In case of disputes, a third party (He-fan He) was consulted to reach a consensus. The extracted contents included (a) the basic information contained in the study, including first author and publication year; (b) the study characteristics, including sample size, patient age, ASIA grade, SCI segment, treatment time, MSC-related information (cell source, cell number, transplantation method, frequency), and follow-up time; (c) outcome measures of interest including the ASIA motor and sensory scores, ASIA grade improvement, BI living ability scores, and incidence of adverse reactions; and (d) relevant elements of the bias risk assessment.

Based on cochrane collaboration’s tool, the methodological quality of included trials, and risk of bias were evaluated by two review authors, which included seven domains: allocation concealment, random sequence generation, incomplete outcome data, selective outcome reporting, blinding of participants, and personnel, blinding of outcome assessment, and other biases [13].

## Statistical analysis

All network meta-analyses (NMAs) and standard meta-analyses were performed using the STATA 14.0 software (Stata Corporation, Texas). The ASIA motor and sensory scores, ASIA grade improvement, BI, and adverse reactions were used as outcome indicators. First, the heterogeneity between the study results was analyzed following the χ^2^ test, and the test level was set to α = 0.1 and combined with I^2^ to quantitatively judge the heterogeneity. When I^2^ < 50% and P > 0.1, the heterogeneity between studies was considered small and the fixed effects model was used for the statistical analysis. If not, then a random model is used. Apparent clinical heterogeneity was processed by subgroup analysis, sensitivity analysis, or descriptive analysis alone.

Continuous variables were expressed as mean difference (MD) or standard MD (SMD) and the binomial distribution as the odds ratio (OR), and their respective 95% confidence intervals (95% CI) were calculated. The adjusted indirect comparisons were performed with MD, OR, and 95% CIs to assess the indirect comparisons of the efficacy and safety of different stem cell sources and different stem cell transplantation approaches [14]. For the ASIA and BI scores and the incidence of adverse reactions in SCI patients, the largest SUCRA scores indicated the best intervention [15, 16].

## Results and discussion

### Included studies

**Fig. 1:**
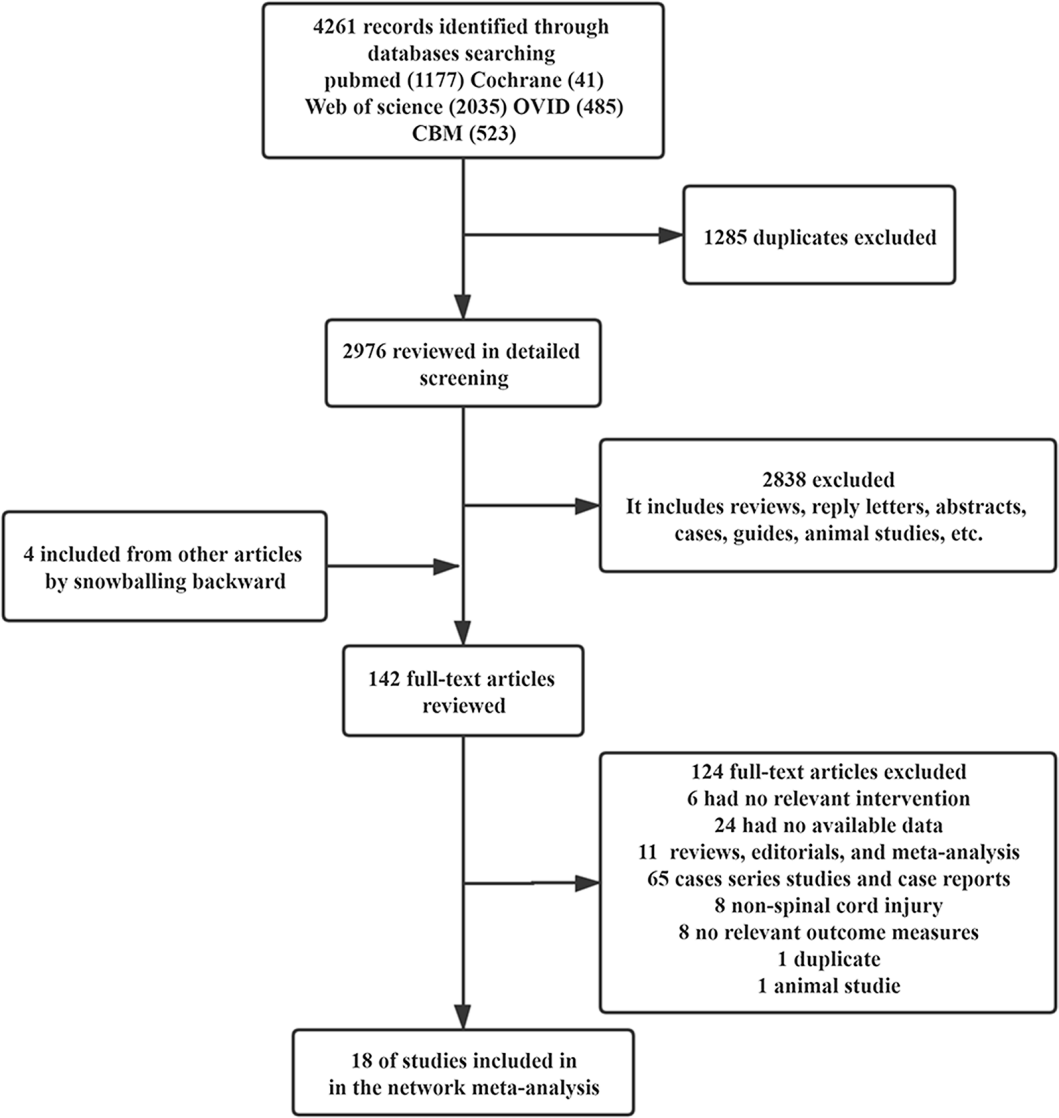
Flow diagram of the trials that were identified and selected

The PUBMED, Cochrane, Web of Science, OVID, and CBM databases were searched and 1177, 41, 2035, 485, and 523 studies were obtained, respectively. After removing the duplicate studies, 2976 studies were retained pending title and abstract screening. Subsequently, 142 records met preliminary criteria and were meticulously reviewed. Finally, 18 studies [17–34] and 949 SCI patients were included in this meta-analysis (Fig. 1).

### Study characteristics

The characteristics of the 18 studies are summarized in Table 1. The sample sizes ranged from 20 to 100. Fifteen studies were conducted in China [17–22, 24–31, 34] and three in Egypt [23, 32, 33]. Of these 949 patients with SCI, 541 were treated with MSCs, and 408 were rehabilitated. The SCI course ranged from hours to years, and follow-up ranged from 1 to 18 months. Eleven studies used autologous MSCs [17, 19, 21, 23–27, 32–34], while the rest used allogeneic MSCs [18, 20, 22, 28, 30, 31]. In addition, a study made a direct comparison of autologous and allogeneic MSCs therapy [29]. Cell transplantation methods included intrathecal (IT), intravenous (IV), and intralesional injection (IL). Two studies were described as "IT and/or IV" [31, 34], and one study did not clearly describe the cell transplantation methods [21]. The frequency of stem cell transplantation treatment ranged from 1 to 6 times and most studies ranged from 1 to 2 times. The clinical outcomes collected included the ASIA motor and sensory scores, BI, and incidence of adverse reactions.

**Table 1:** Characteristics of included studies

Author(s)	Year	Sample size	Injury level (C/T/L)	Treatment/control	M/F	Age (years)	ASIA(A/B/C/D)	Duration	Cell source	Way of transplantation	Cell number	Transplantation times	Follow up (months)	Outcomes
Song et al. [17]	2020	36	10/4/4	18/18	12/6	41.2 ± 2.3	–	–	Auto, bone marrow	IT	2.0 × 10^7^	2	12	①②④
			11/5/2		10/8	41.7 ± 2.1								
Zhang et al. [18]	2019	100	0/42/8	50/50	30/20	41.26 ± 9.74	0/16/20/14	6.55 ± 2.43 h	Allo, umbilical cord	IT	(3–4) × 10^7^/ml	4–6	3,6,12	①②④⑤
			0/44/6		28/22	42.89 ± 10.30	0/15/22/13	6.80 ± 2.66 h						
Tang et al. [19]	2016	60	0/13/17	30/30	21/9	38.2 ± 6.7	–	–	Auto, bone marrow	IT	1 × 10^6^	1	12.4 ± 2.3	①②③
			0/14/16		20/10	37.9 ± 7.2								
Zhang et al. [20]	2015	30	10/5/0	15/15	11/4	35.5 ± 8.3	9/3/3/0	21.3 ± 5.7 months	Allo, umbilical cord	IL	8 × 10^7^	2	6	①②④
			10/5/0		11/4	35.7 ± 8.3	–	19.7 ± 7.6 months						
Zhang et al. [21]	2015	100	–	50/50	16/34	36.4 ± 1.9	–	–	Auto, bone marrow	–	(2–4) × 10^2^ /kg	–	6	①②③
					15/35	37.3 ± 2.0								
Cheng et al. [22]	2014	20	–	10/10	–	35.30 ± 8.23	10/0/0/0	21.40 ± 12.96 months	Allo, umbilical cord	IL	4 × 10^7^	2	6	①②③④
						36.64 ± 9.90	10/0/0/0	18.57 ± 11.35 months						
El-kheir et al. [23]	2014	70	10/40/0	50/20	61/9	16–45	15/35/0/0	18.3 ± 5.0 months	Auto, bone marrow	IT	2 × 10^6^ cells/kg	–	18	①②⑤
			7/13/0				10/10/0/0							
Guo et al. [24]	2014	80	17/3/20	40/40	30/10	36.4 ± 1.9	–	> 1 month	Auto, bone marrow	IL	(2–4) × 10^2^/kg	2	3	①②③④
			13/3/24		33/7	37.3 ± 2.0								
Xiao et al. [25]	2014	64	7/12/16	35/29	23/12	42.8 ± 10.2	–	22.7 ± 5.4 days	Auto, bone marrow	IT	(8.47 ± 3.54) × 10^7^	2	6	①②④
			6/10/13		19/10	41.4 ± 10.5								
Dai et al. [26]	2013	40	20/0/0	20/20	14/6	34.7 ± 8.9	20/0/0/0	51.9 ± 18.3 months	Auto, bone marrow	IL	8 × 10^5^	1	6	①②④⑤
			20/0/0		14/6	35.1 ± 8.0	20/0/0/0	43.2 ± 15.3 months						
Xiao et al. [27]	2012	96	7/15/16 (IT)	38/32/26	25/13	42.3 ± 10.2	–	25.2 ± 6.7 days	Auto, bone marrow	IT and IV	(13.58 ± 4.62) × 10^6^	1	6	①②④
			6/12/14(IV)		21/11	41.5 ± 10.7					(13.58 ± 4.62) × 10^6^			
			5/9/12 (Re)		17/09	41.2 ± 10.6								
Guo et al. [28]	2012	24	–	12/12	11/1	29	–	2.3–2.5 months	Allo, umbilical cord	IT	(2–5) × 10^7^	4	6	①②③
					10/2	31								
Dai et al. [29]	2012	23	10/11/2	15/8 (Auto/Allo)	16/7	28.22 ± 16.19	–	1–72 months	Auto, bone marrow	IT	1–1.5 × 10^8^↑	4–6	3	①②
									Allo, umbilical cord					
Zhang et al. [30]	2012	60	12/20/28	30/30	50/10	32.5 ± 4.2	–	1–10 months	Allo, umbilical cord	IV	3 × 10^8^	1	3	①②③④
Li [31]	2012	30	5/8/1	14/15	11/3	37.36 ± 11.06	8/4/3/0	1 month–9 years	Allo, umbilical cord	IV and IT	5 × 10^7^	–	4	①②③④
			4/10/1		10/5	37.67 ± 11.84	7/4/4/0							
Kishk et al. [32]	2010	64	6/37/0	43/20	36/7	31.7 ± 10.4	–	3.6 ± 2.5 years	Auto, bone marrow	IT	3.75 × 10^8^ to	6	6	①②④⑤
			2/18/0		15/5	33.8 ± 11.8		3.7 ± 2.1 years			7.5 × 10^8^			
Abdelaziz et al. [33]	2010	30	0/20/0	20/10	18/2	6.0–52.0	–	27.2 months	Auto, bone marrow	IL	5 million cells/kg	6	12	④⑤
			0/10/0		07/3	6.0–64.0								
Xie et al. [34]	2007	24	2/4/5	11/13	9/2	18.0–49.0	8/0/1/2	1–12 months	Auto, bone marrow	IT or IV	(4.87–8.8) × 10^7^	3	3	①③④⑤
			3/4/6		10/3	21.0–53.0	9/1/2/1							

C/T/L: cervical/thoracic/lumbar spinal cord; M/F, male/female; Auto, autologous mesenchymal stem cells; Allo, allogeneic mesenchymal stem cells; Re, rehabilitation; IT, intrathecal injection; IL, intralesional injection; IV, intravenous injection; Re, rehabilitation. ① American Spinal Injury Association Motor Score, ② American Spinal Injury Association Sensory Score, ③ Barthel index, ④adverse reactions, ⑤ASIA grade improvement

### Methodologic quality and risk of bias

**Fig. 2:**
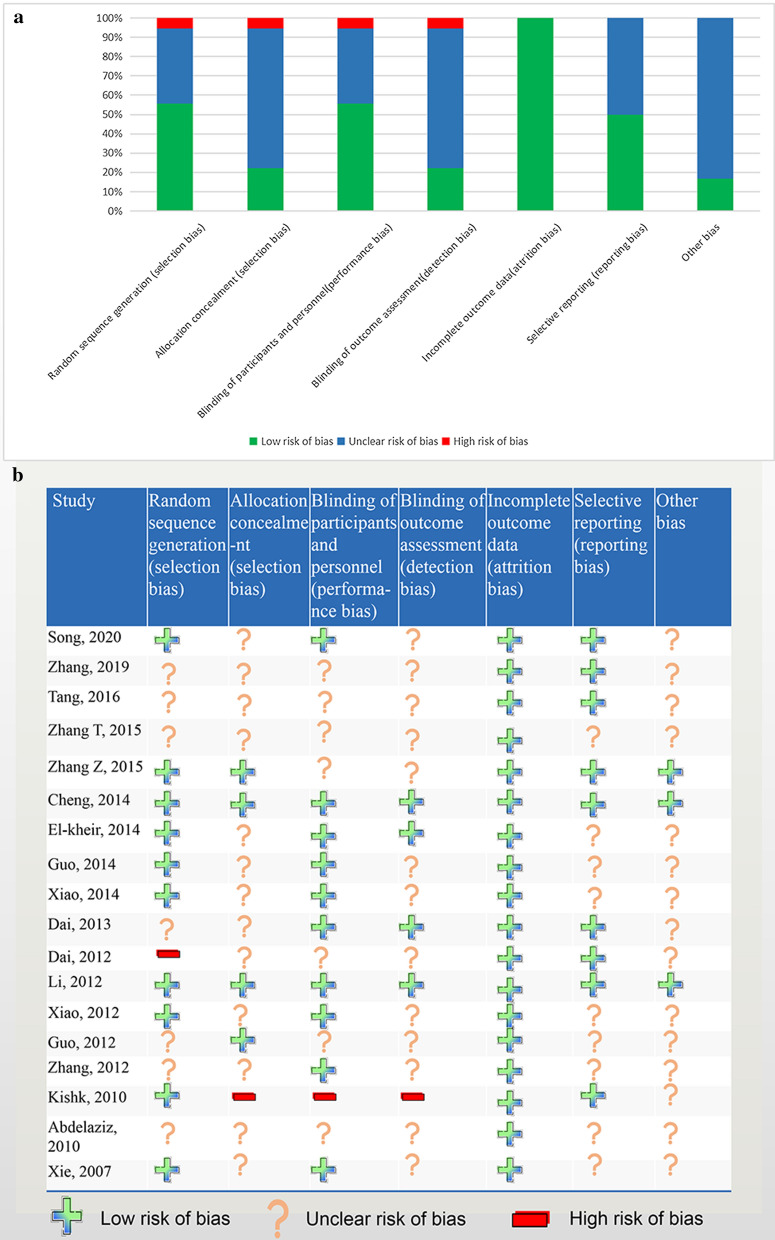
Risk of bias. **a** Risk of bias graph. **b** Risk of bias summary

We used the standard Cochrane collaborative tool to assess the risk of bias in the included studies, and the methodological quality results for each trial are shown in Fig. 2. Ten studies reported a generation of random sequences, while one study determined the treatment according to the patient's condition, while the other seven studies did not mention their rationale for selection. No studies reported using the allocation concealment. Moreover, four studies had outcome blinding details, one study had no outcome blinding, and other studies did not indicate the outcome blinding. Most of the studies had no missing data and only two studies had missing data, but the reasons for missing data were reported in the study. None of the studies selectively reported the results or other biases. Overall, the methodological quality of the included studies was acceptable.

### Standard meta-analysis

#### ASIA motor and sensory scores

**Fig. 3:**
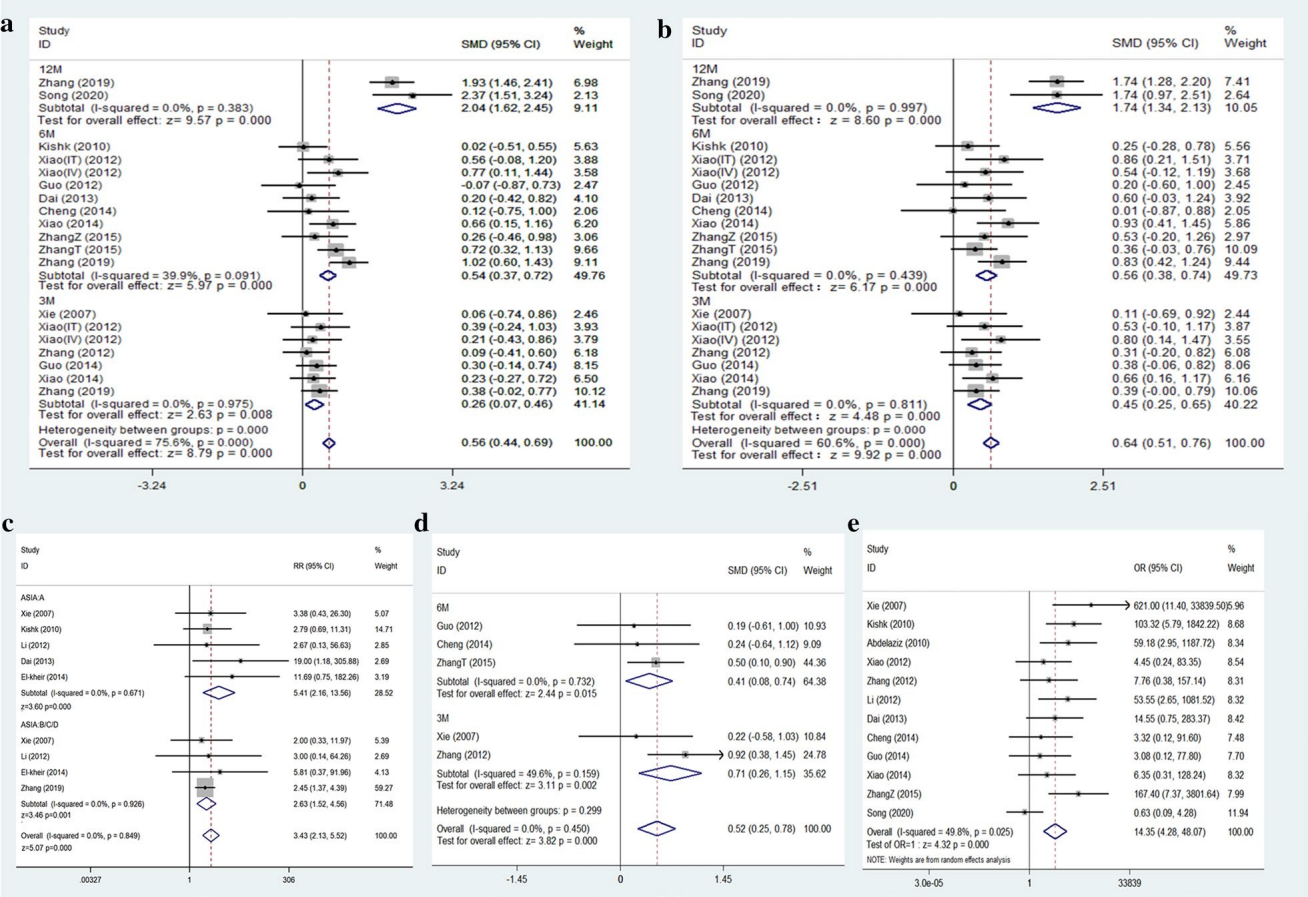
Forest plot of comparison: MSC transplantation group versus control group. **a** ASIA motor score; **b** ASIA sensory score; **c** ASIA grade improvement; **d** Barthel index; **e** adverse effects. ASIA: American Spinal Injury Association; CI: confidence interval; SMD: standard mean difference

Thirteen articles comprising of 19 studies reported ASIA motor and sensory scores in two different groups at different follow-up times. Due to the large overall heterogeneity (ASIA motor score: I^2^ = 75.6%, P < 0.001; ASIA sensory score: I^2^ = 60.6%, P < 0.001), they were further divided into three subgroups according to the follow-up time (3 months, 6 months, and 12 months). The I^2^ of each subgroup included was less than 50%, indicating low heterogeneity (Fig. 3a, b), so a fixed-effect model was used for meta-analysis of the ASIA motor and sensory scores. The analysis results revealed that MSC transplantation increases the ASIA motor and sensory scores compared to rehabilitation in SCI patients [ASIA motor score: 12 months: SMD = 2.04, 95% CI (1.62, 2.45), P < 0.001; 6 months: SMD = 0.54, 95% CI (0.37, 0.72), P < 0.001; 3 months: SMD = 0.26, 95% CI (0.07, 0.46), P < 0.01, Fig. 3a; ASIA sensory score: 12 months: SMD = 1.74, 95% CI (1.34, 2.13), P < 0.001; 6 months:SMD = 0.56, 95% CI (0.38, 0.74), P < 0.001; 3 months: SMD = 0.45, 95% CI (0.25, 0.65), P < 0.001, Fig. 3b]. After excluding the studies included in the sensitivity analysis, the merged results did not change significantly, indicating that the results were robust (Additional file 1: Figure S1).

#### ASIA grade improvement

Six articles included nine studies reporting the improvement of SCI patients with different ASIA grades (Grade A or B/C/D) after MSCs treatment. Since the overall heterogeneity of the included studies was not significant (I^2^ = 0.0%, P = 0.849), a fixed-effects model was used for meta-analysis. The results showed that compared with the control group, the treatment group had a significant improvement in ASIA grade A and grade B/C/D [ASIA A: RR = 5.41, 95% CI (2.16, 13.56), P < 0.001; ASIA B/C/D: RR = 2.63, 95% CI (1.52, 4.56), P < 0.05 Fig. 3c].

#### Barthel index

Six of the included studies reported BI. Upon conducting the sensitivity analysis, one study had a greater impact on the overall effect size [24] and therefore was excluded from the BI meta-analysis (Additional file 1: Figure S1). The remaining five studies had low overall heterogeneity (I^2^ < 30%, P = 0.45), so a fixed-effect model was used for BI's meta-analysis. The results showed that MSCs transplantation increased BI [SMD = 0.52, 95% CI (0.25, 0.78), P < 0.001, Fig. 3d] compared to rehabilitation. The patients were further divided into two subgroups according to the follow-up time (3 months and 6 months). The I^2^ of the included subgroups was less than 50%, indicating a low heterogeneity (Fig. 3d). Meta-analysis using the fixed-effect model confirmed that MSC transplantation was superior to the rehabilitation for improvement in BI [6 months: SMD = 0.41, 95% CI (0.08, 0.74), P < 0.05; 3 months: SMD = 0.71, 95% CI (0.26, 1.15), P < 0.005, Fig. 3d].

#### Adverse effects

Thirteen studies reported complications during treatment and follow-up, all reporting no severe complications, tumors, or abnormal tissue proliferation. Common adverse reactions included fever, headache, back pain, and numbness. These symptoms could be alleviated by themselves or after symptomatic treatment. After excluding one trial from the sensitivity analysis, the meta-analysis results showed that the adverse reactions of patients with mesenchymal stem cell transplantation were greater than that of the control group [OR = 14.35, 95% CI (4.28, 48.07), P < 0.001, Fig. 3e].

## Network meta-analysis

### Comparison between different cell sources

#### Network plot

**Fig. 4:**
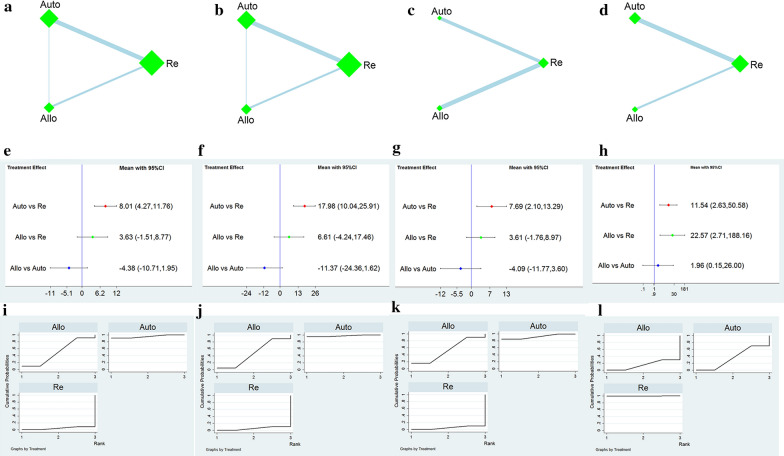
Network meta-analysis of different cell sources. **a**–**d** Network plot of the subgroup; **e**–**h** Forest plot represents the direct and indirect comparison; **i**–**l** the surface under the cumulative ranking curves for different outcomes. From left to right are ASIA motor score, ASIA sensory score, Barthel index, and adverse reactions, respectively. Auto: autologous mesenchymal stem cells, Allo: allogeneic mesenchymal stem cells, Re: rehabilitation

We generated four networks for the four primary outcomes. Each network plot involved different sources of MSCs. The summarized network plots of the comparisons are shown in Fig. 4a–d.

#### ASIA motor and sensory scores

Outcome measures for one study did not mention the ASIA motor and sensory scores and were thereby excluded [33]. One study was a 3-arm study [27], and the other was divided into two subgroups [23]; therefore, 19 pairwise comparisons were included. The results of the NMAs revealed that the ASIA motor [MD = 8.01, 95% CI (4.27, 11.76)] and sensory scores [MD = 17.98, 95% CI (10.04, 25.91)] improved significantly by the autologous mesenchymal stem cell therapy compared to the rehabilitation therapy. However, no significant difference was observed in other comparisons (Fig. 4e, f).

#### Barthel index

The outcome measures for seven eligible studies included BI. The NMAs showed that autologous MSC transplantation significantly improved BI compared with rehabilitation in patients with SCI [MD = 7.69, 95% CI (2.10, 13.29)]. In contrast, no significant differences were observed in other comparisons (Fig. 4g).

#### Adverse reactions

After excluding one study with significant heterogeneity based on the sensitivity analysis [18], a total of 12 of the included studies reported adverse reactions. The NMAs revealed no significant difference in the adverse reactions between autologous and allogeneic MSC transplantation; however, both autologous and allogeneic MSCs had more adverse reactions compared to rehabilitation [OR = 11.54, 95% CI (2.63, 50.58); OR = 22.57, 95% CI (2.71, 188.16), Fig. 4h].

#### Ranking probability

The MSCs ranking from different sources is shown in Table 2. The ranking of the ASIA motor, sensory score, and BI from high to low is autologous MSCs (95.4%, 97.8%, and 92.4%), allogeneic MSCs (50.4%, 46.9%, 52.6%) and rehabilitation (4.3%, 5.3%, and 5.0%); fewer adverse reactions ranked from high to low is as follows: rehabilitation (99.9%), autologous MSCs (34.4%), and allogeneic MSCs therapy (15.7%). The SUCRA ranking map was constructed according to the SUCRA curve (Fig. 4i–l).

**Table 2:** Ranking for all the interventions’ outcomes in the network meta-analysis

	Interventions	ASIA motor score			ASIA sensory score			Barthel index			Adverse reactions		
		SUCRA(%)	PrBest	Mean rank	SUCRA(%)	PrBest	Mean rank	SUCRA(%)	PrBest	Mean rank	SUCRA(%)	PrBest	Mean rank
Cell sources	Re	4.3	0	2.9	5.3	9	2.9	5	0	2.9	99.9	99.8	1
	Auto	95.4	90.8	1.1	97.8	95.6	1	92.4	85.2	1.2	34.4	0.1	2.3
	Allo	50.4	9.2	2	46.9	4.4	2.1	52.6	14.8	1.9	15.7	0.1	2.7
Cell transplantation ways	Re	7.8	0	3.8	13.6	0	3.6	19.1	0.6	3.4	91.4	75.8	1.3
	IT	85.5	60.2	1.4	91.2	75.8	1.3	61.4	24.5	2.2	46.7	6.9	2.6
	IL	38.3	4.9	2.9	42.3	6.4	2.7	44	21.8	2.7	19.7	0.1	3.4
	IV	68.4	34.9	1.9	52.9	17.8	2.4	75.5	53.1	1.7	42.3	17.2	2.7

*SUCRA* surface under the cumulative ranking, *PrBest* the probability of best treatment, *Auto* Autologous mesenchymal stem cells, *Allo* allogeneic mesenchymal stem cells, *Re* rehabilitation, *IT* intrathecal injection, *IL* intralesional injection, IV intravenous injection

### Comparison between different transplant ways

#### Network plot

**Fig. 5:**
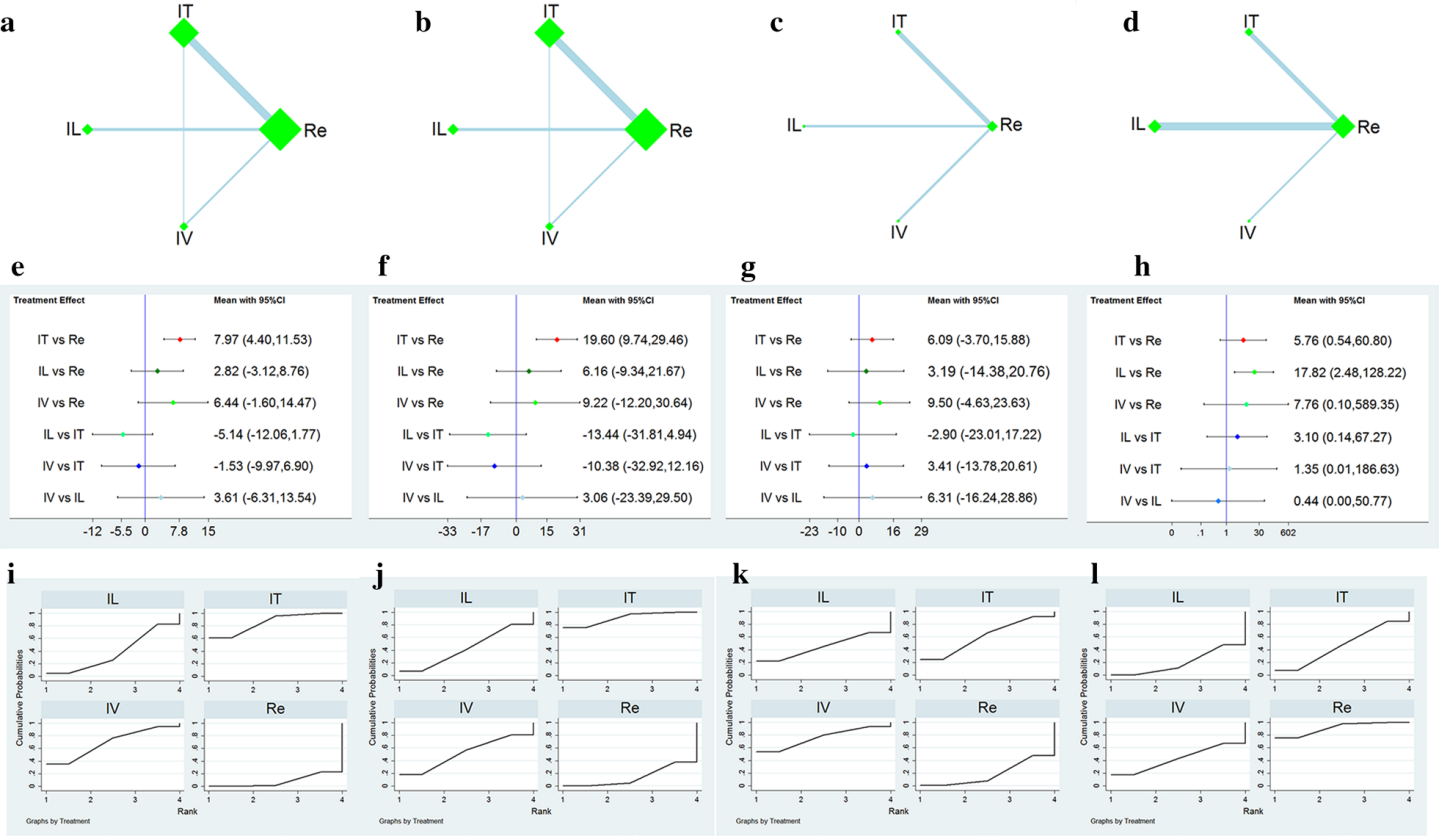
Network meta-analysis of different cell transplantation ways. **a**–**d** Network plot of the subgroup; **e**–**h** Forest plot represents the direct and indirect comparison; **i**–**l** The surface under the cumulative ranking curves for different outcomes. From left to right are ASIA motor score, ASIA sensory score, Barthel index, and adverse reactions, respectively. IT: intrathecal injection, IL: intralesional injection, IV: intravenous injection, Re: rehabilitation

We generated four network maps containing the four outcome measures. Each network plot has a different stem cell transplantation method, namely the Re, IT, IL, and IV. Figure 5 (a–d) provides a summary network map for comparison.

#### ASIA motor and sensory scores

Two studies were described as "IT and/or IV" [31, 34]. One study did not clearly define the method of stem cell transplantation [21] and another study excluded the ASIA motor and sensory scores as the outcome measures [27]. One study was a 3-arm study [23] and the other was divided into two subgroups, therefore containing 16 pairwise comparisons. The NMAs revealed no significant differences in ASIA motor and sensory scores among the three cell transplantation approaches, as shown in Fig. 5e, f. Compared to rehabilitation, only IT improved the ASIA motor [MD = 7.97, 95% CI (4.40, 11.53), Fig. 5e] and sensory scores [MD = 19.60, 95% CI (9.74, 29.46), Fig. 5f].

#### Barthel index

Four studies that met the criteria included BI as an outcome measure. The results showed that there was no significant difference in the pairwise comparison between the four intervention methods (Fig. 5g).

#### Adverse reactions

After sensitivity analysis excluded one trial with considerable heterogeneity [18], a total of 9 trials included in the study of different stem cell transplantation methods reported adverse reactions. The NMAs results showed no significant difference in the adverse reactions between the different transplantation approaches of MSCs. Compared to rehabilitation, however, only the IL of MSCs was associated with more adverse reactions [OR = 17.82, 95% CI (2.48, 128.22); Fig. 5h].

#### Ranking probability

The ranking of different stem cell transplantation methods is shown in Table 2. The ranking of ASIA motor and sensory score from high to low is IT (85.5%, 91.2%), IV (68.4%, 52.9%), IL (38.3%, 42.3%), and rehabilitation (7.8%, 13.6%); the ranking of BI from high to low is as follows: IV (75.5%), IT (61.4%), IL (44.0%) and rehabilitation treatment (19.1%); the ranking of mild adverse reactions from high to low is as follows: rehabilitation (91.4%), IT (46.7%), IV (42.3%), and IL (19.7%). The SUCRA ranking graph was erected according to the SUCRA curve (Fig. 5i–l).

### Inconsistency analysis

Inconsistency refers to the difference between direct and indirect evidence, which affects the authenticity of NMAs. We used the relative odds ratio (ROR) with 95% CI to calculate the absolute difference between the direct and indirect evidence. If the ROR is close to 1, or 95% CI contains 0, the effect estimates of direct and indirect evidence are consistent. No closed loop was formed in BI and adverse reaction outcome measures; therefore, no inconsistency analysis was conducted. However, no significant inconsistency was observed in the resulting closed-loop comparing ASIA motor and sensory scores from different cell sources and transplantation methods, suggesting that the consistency model's conclusions were robust (Additional file 1: Figure S2).

### Publication bias and sensitivity analysis

Since fewer studies included the BI outcome measures, publication bias could not be explored. The funnel plot for the adjustment of all results in other NMAs is presented in Additional file 1: Figure S3. The funnel plot indicated that these results were not entirely symmetrical, possibly due to the small sample size or publication bias. Nonetheless, sensitivity analysis confirmed that there were no trials with a high risk of bias (Additional file 1: Figure S1).

## Discussion

This study investigated the efficacy and safety of MSCs transplantation in SCI treatment. Our study confirms that MSCs transplantation significantly improves neurological function, including the ASIA motor, sensory, ASIA grade improvement, and BI, compared to rehabilitation therapy. However, some mild and temporary side effects occur in patients that receive MSCs transplantation.

To compare the different cell sources and transplantation methods of MSCs, NMAs were used to compare the indirect evidence in the meta-analysis. We found that transplantation of all autologous MSCs was more effective than rehabilitation in ASIA motor, sensory, and BI. Consistent with the standard meta-analysis, both autologous and allogeneic MSCs transplantation patients had some reversible adverse reactions. In the ranking probability of effectiveness and safety, autologous MSCs transplantation was better than the allogeneic MSCs transplantation.

Moreover, compared to rehabilitation, only IT transplantation of MSCs significantly improved the ASIA motor and sensory scores. However, the toxicity of IL-transplanted MSCs is higher than that of rehabilitation therapy. In the ASIA motor, sensory, and safety evaluation, IT transplantation stem cells ranked first, followed by IV and IL. In BI, however, the IV transplantation stem cells ranked first, followed by IT and IL.

In preclinical studies, MSCs transplantation has several advantages in the treatment of SCI. MSCs exosomes exert immunomodulatory, anti-inflammatory, neurotrophic/neuroprotective, and angiogenic effects on the host microenvironment [35]. MSCs not only perform an immunomodulatory role by inhibiting the activation, proliferation, and differentiation of T cells [36], but also play an anti-inflammatory role by secreting a variety of soluble factors, such as the tumor necrosis factor (TNF)-β1, interleukin (IL)-10, IL-27, and neurotrophic factor 3 (NT-3) [37]. Furthermore, as a neuroprotective role, MSCs secrete many neurotrophic factors, such as brain-derived growth factor (BDNF), glial-derived growth factor (GDNF), nerve growth factor (NGF), NT-1, NT-3, and basic fibroblast growth factor (bFGF) [10, 35]. It is worth noting that some studies have shown that MSCs can survive and differentiate into different cell types, including neurons, oligodendrocytes, and astrocytes [38].

MSCs can be collected from autologous bone marrow, adipose tissue, and allogeneic umbilical cord [39]. For a long time, MSCs have been reported as low immunogenic or immune-privileged [40]. However, recent studies have described the antibody production and immune rejection against allogeneic MSCs, suggesting that MSCs may not be immune-privileged [41]. Although it is not clear whether MSC rejection affects the efficacy of allogeneic MSC therapy, protecting MSCs from immune response and prolonging its persistence in vivo can improve the clinical outcomes and prevent sensitivity to donor antigens [42]. Indeed, our NMAs indicate that autologous MSCs rank better than the allogeneic MSCs in terms of efficacy and safety. Therefore, autologous MSCs may be the most suitable cell source for SCI treatment. However, this conclusion comes from the indirect comparison results. To exclude the effect of transplanted cell volume, frequency, duration, and severity of SCI, further well-designed and high-quality clinical, randomized controlled trials are required.

MSCs are transplanted into patients with SCI through the IT, IL, and IV routes. However, different transplantation methods may be one of the reasons that affect the efficacy of MSCs. In animal experiments stem cells do not significantly improve nerve function [43], as the IL of stem cells may cause secondary injury to the spinal cord. Our NMAs also confirmed that in SCI patients, the adverse effects of IL transplantation of MSCs were significantly increased compared to the rehabilitation treatment. For IV, the transplanted MSCs migrate through the brain spinal cord barrier to the spinal cord under the lesion's chemokines [35]. Nevertheless, most of the cells transplanted through IV are trapped in the lung, and only a small proportion of transplanted cells migrate to the lesion site, which significantly reduces the plantation rate of stem cells [39]. Furthermore, Shin et al. considered that direct injection of stem cells into cerebrospinal fluid may be the safest and most effective method for cell transplantation in SCI [44]. Consistent with the SUCRA curve, our results show that IT transplantation of MSCs in SCI treatment is superior to IL and IV transplantation in terms of ASIA motor, sensory score, and the incidence of adverse reactions. Therefore, IT may be a fitting method for transplantation. However, in the future, it will be necessary to carry out a comparative study on the efficacy of different MSCs transplantation approaches in the treatment of SCI to elucidate the optimal stem cell transplantation method.

As for the efficacy and safety of MSCs in SCI treatment, the previous meta-analysis results were similar to those in this study [11]. However, it is unclear whether the MSCs are from autologous or allogeneic sources and the effects of different cell transplantation approaches. In contrast, our study has the following advantages: First, we used the ASIA motor and sensory scores as continuous variables to exclude the grouping errors. Second, we defined the source of cells and the method of transplantation. Finally, we adopted NMAs to rank the subgroups from various cell sources that could not be directly compared, and the best way of cell transplantation was investigated using the indirect comparison.

However, this study has several limitations. (a) The efficacy evaluation index was not sufficiently comprehensive. Since most of the included studies did not report urodynamics and muscle tone measures, only the ASIA and BI scores were used as effect measurements. (b) The quality of the included studies was uneven and many trials did not clearly describe the design of randomization, whether they used allocation concealment and blindness, so it is possible to overestimate the efficacy of MSCs transplantation for SCI. (c) The original studies’ data were limited, so we were unable to analyze the therapeutic effects of different MSCs in terms of size, transplantation time, SCI grade, and course of the disease. (d) Although autologous MSCs and IT are considered appropriate cell sources and transplantation methods by the NMAs, the number of studies between the two direct comparisons is small. Consequently, although the results of this meta-analysis are robust, caution should be exercised in interpreting the results due to limited data.

## Conclusion

In SCI, MSCs transplantation generates better outcomes than rehabilitation, including improvements in movement, sensation, and quality of life. For indirect head-to-head comparisons, there were no significant differences when comparing the different cell sources and transplantation methods. Nevertheless, the treatment of SCI by IT transplantation of autologous MSCs may be a better option. However, further clinical head-to-head trials are required to confirm the effectiveness and safety of these interventions.

## Supplementary Information

**Additional file 1:** **Table S1**: Search strategy. **Figure S1**. sensitivity analysis. **a** ASIA motor score; **b** ASIA sensory score; **c** Barthel index; **d** Adverse effects. **Figure S2**. Inconsistency in closed loops for all outcomes. **a**–**c** Network meta-analysis of different cell sources. **d**–**f** Network meta-analysis of different cell transplantation methods. From left to right are the ASIA motor score, ASIA sensory score, and adverse reactions, respectively.

## Data Availability

The data used to support the findings of this study are available from the corresponding author upon request.
