# Supplementary material for: Transplantation of mesenchymal stem cells for spinal cord injury: a systematic review and network meta-analysis
Source: J Transl Med. 2021 Apr 28;19:178. doi: 10.1186/s12967-021-02843-0 (PMC8082850; doi:10.1186/s12967-021-02843-0)

### supplementary materials

**Supplementary Table 1**. Search strategy.

| Data source | Search terms | Total |
| --- | --- | --- |
| Pubmed | (((((((((((((((((((((((((((((((((((((((("Spinal Cord Injuries"[Mesh]) ) OR (Spinal Cord Trauma)) OR (Cord Trauma, Spinal)) OR (Cord Traumas, Spinal)) OR (Spinal Cord Traumas)) OR (Trauma, Spinal Cord)) OR (Traumas, Spinal Cord)) OR (Myelopathy, Traumatic)) OR (Myelopathies, Traumatic)) OR (Traumatic Myelopathies)) OR (Traumatic Myelopathy)) OR (Injuries, Spinal Cord)) OR (Cord Injuries, Spinal)) OR (Cord Injury, Spinal)) OR (Injury, Spinal Cord)) OR (Spinal Cord Injury)) OR (Spinal Cord Transection)) OR (Cord Transection, Spinal)) OR (Cord Transections, Spinal)) OR (Spinal Cord Transections)) OR (Transection, Spinal Cord)) OR (Transections, Spinal Cord)) OR (Spinal Cord Laceration)) OR (Cord Laceration, Spinal)) OR (Cord Lacerations, Spinal)) OR (Laceration, Spinal Cord)) OR (Lacerations, Spinal Cord)) OR (Spinal Cord Lacerations)) OR (Post-Traumatic Myelopathy)) OR (Myelopathies, Post-Traumatic)) OR (Myelopathy, Post-Traumatic)) OR (Post Traumatic Myelopathy)) OR (Post-Traumatic Myelopathies)) OR (Spinal Cord Contusion)) OR (Contusion, Spinal Cord)) OR (Contusions, Spinal Cord)) OR (Cord Contusion, Spinal)) OR (Cord Contusions, Spinal)) OR (Spinal Cord Contusions)) AND (((((((((((((((((((((((((((((((((((Mesenchymal Stem Cells[Mesh]) ) OR (Stem Cell, Mesenchymal)) OR (Stem Cells, Mesenchymal)) OR (Mesenchymal Stem Cell)) OR (Bone Marrow Mesenchymal Stem Cells)) OR (Bone Marrow Stromal Cells)) OR (Bone Marrow Stromal Cell)) OR (Bone Marrow Stromal Cells, Multipotent)) OR (Multipotent Bone Marrow Stromal Cells)) OR (Adipose-Derived Mesenchymal Stem Cells)) OR (Adipose Derived Mesenchymal Stem Cells)) OR (Mesenchymal Stem Cells, Adipose-Derived)) OR (Mesenchymal Stem Cells, Adipose Derived)) OR (Adipose-Derived Mesenchymal Stromal Cells)) OR (Adipose Derived Mesenchymal Stromal Cells)) OR (Adipose Tissue-Derived Mesenchymal Stem Cells)) OR (Adipose Tissue Derived Mesenchymal Stem Cells)) OR (Adipose Tissue-Derived Mesenchymal Stromal Cells)) OR (Adipose Tissue Derived Mesenchymal Stromal Cells)) OR (Mesenchymal Stromal Cells)) OR (Mesenchymal Stromal Cell)) OR (Stromal Cell, Mesenchymal)) OR (Stromal Cells, Mesenchymal)) OR (Multipotent Mesenchymal Stromal Cells)) OR (Mesenchymal Stromal Cells, Multipotent)) OR (Mesenchymal Progenitor Cell)) OR (Mesenchymal Progenitor Cells)) OR (Progenitor Cell, Mesenchymal)) OR (Progenitor Cells, Mesenchymal)) OR (Wharton Jelly Cells)) OR (Wharton's Jelly Cells)) OR (Wharton's Jelly Cell)) OR (Whartons Jelly Cells)) OR (Bone Marrow Stromal Stem Cells)) | 1177 |
| Cochrane | Spinal Cord Injuries and Mesenchymal Stem Cells | 41 |
| Web of science | **TOPIC**: (Spinal Cord Injuries and Mesenchymal Stem Cells)  Databases= WOS, KJD, MEDLINE, RSCI, SCIELO Timespan=All years  Search language=Auto | 2035 |
| OVID | (Spinal Cord Injuries and Mesenchymal Stem Cells).mp. [mp=title, abstract, original title, name of substance word, subject heading word, floating sub-heading word, keyword heading word, organism supplementary concept word, protocol supplementary concept word, rare disease supplementary concept word, unique identifier, synonyms] | 485 |
| CBM | Spinal cord injury [intelligence] AND Mesenchymal stem cells [intelligence] | 523 |
| Total | up to [April](../../../../D:/LenovoSoftstore/Install/wangyiyoudaocidian/8.9.6.0/resultui/html/index.html#/javascript:;) 01, 2021 | 4261 |

**Supplementary Figure 1. sensitivity analysis.** (A) ASIA motor score; (B) ASIA sensory score; (C) Barthel Index; (D) adverse effects.


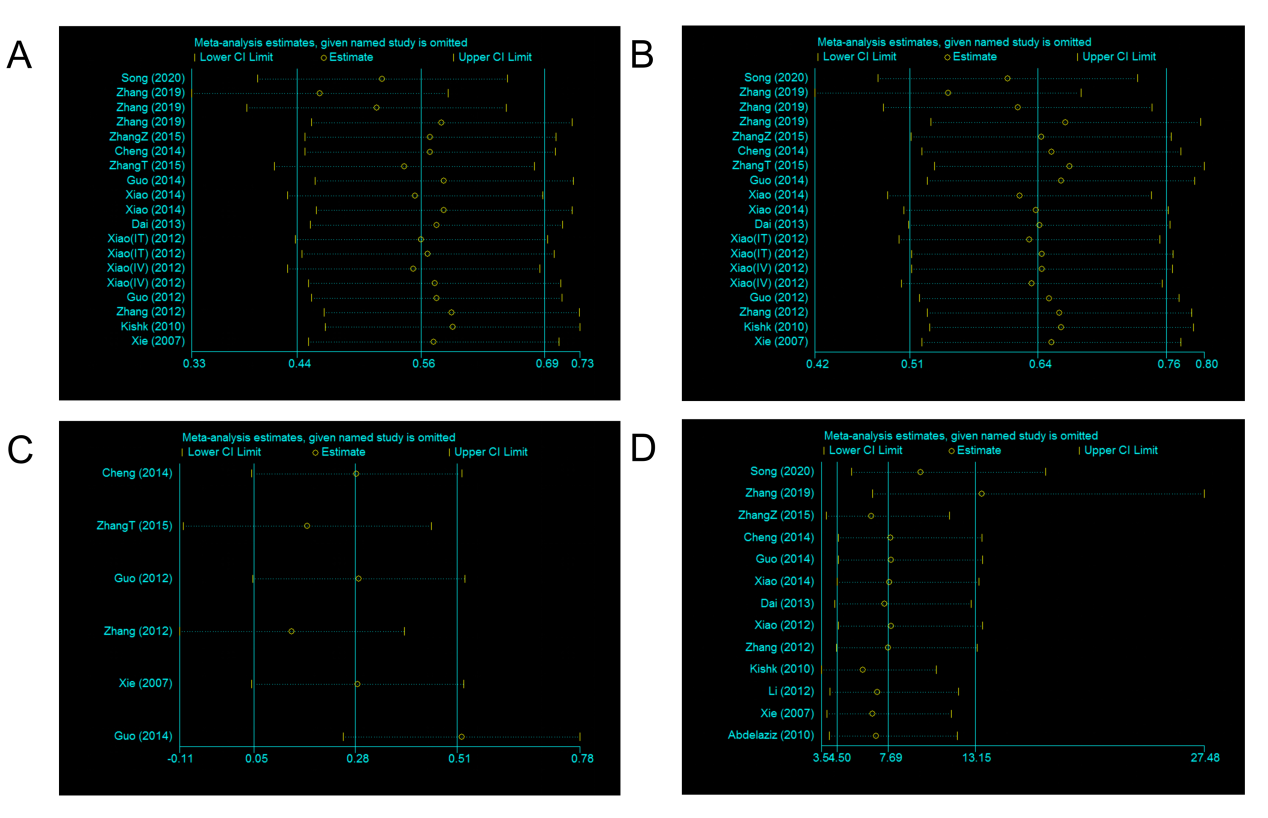


**Supplementary Figure 2. Inconsistency in closed loops for all the outcomes**. （A-C）Network meta-analysis of different cell sources. (D-F)Network meta-analysis of different cell transplantation ways. From left to right are ASIA motor score, ASIA sensory score, and adverse reactions, respectively.


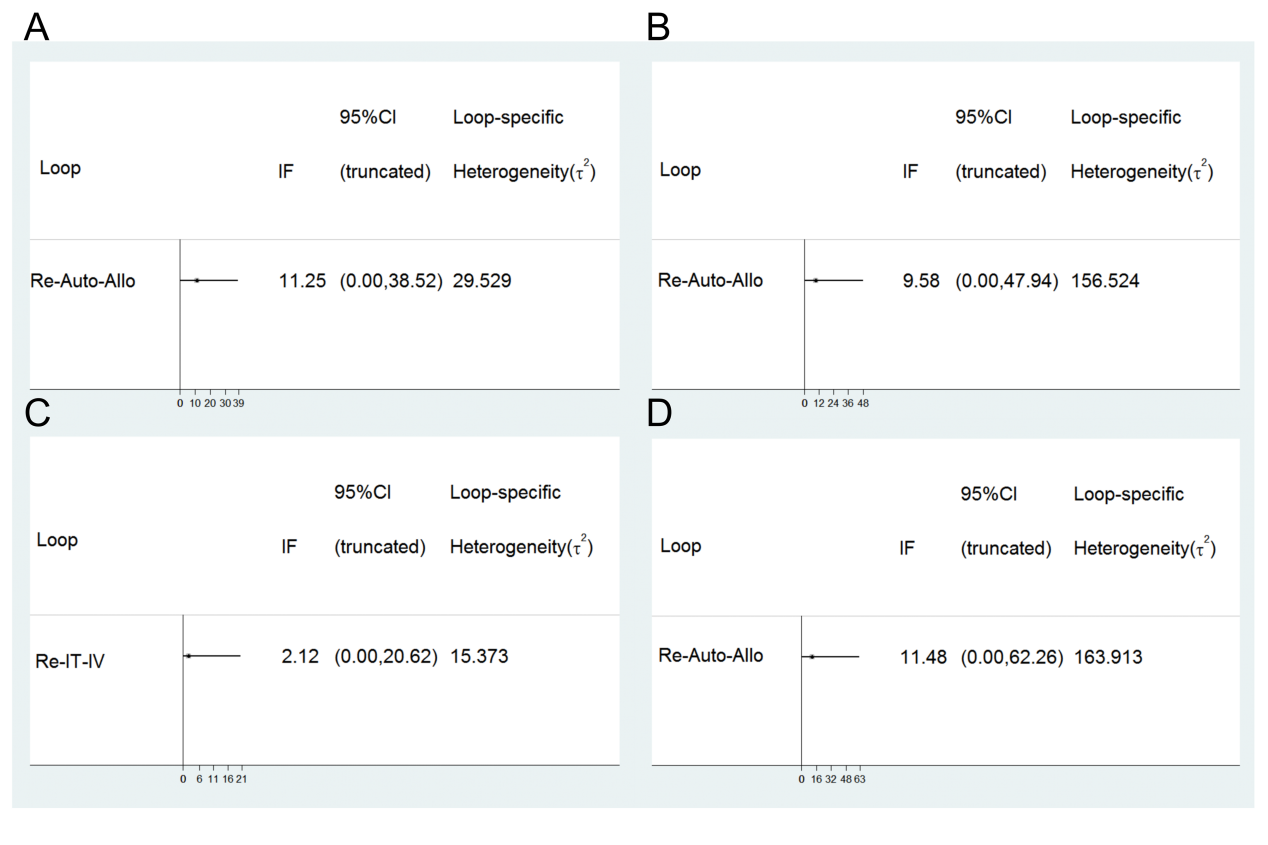


**Supplementary Figure 3. funnel plot.**（A-C）Network meta-analysis of different cell sources. (D-F)Network meta-analysis of different cell transplantation ways. From left to right are ASIA motor score, ASIA sensory score, and adverse reactions, respectively.


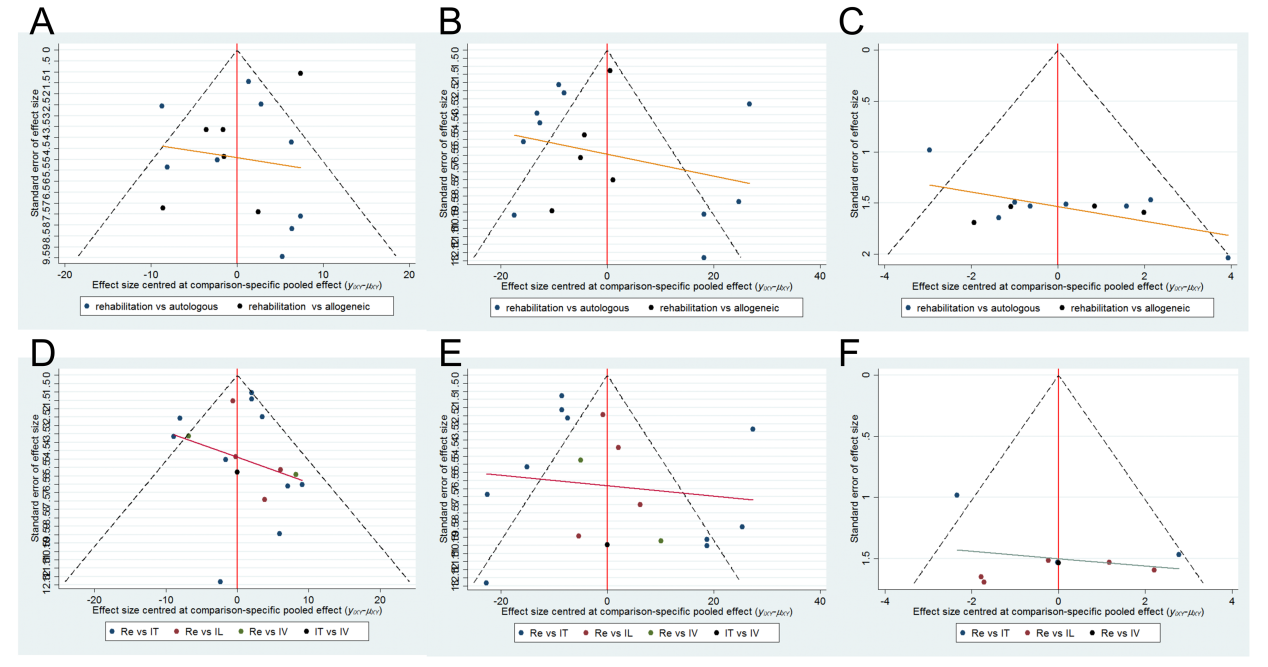

Supplement: Supplementary file 1 — Additional file 1: Table S1: Search strategy. Figure S1. sensitivity analysis. a ASIA motor score; b ASIA sensory score; c Barthel index; d Adverse effects. Figure S2. Inconsistency in closed loops for all outcomes. a–c Network meta-analysis of different cell sources. d–f Network meta-analysis of different cell transplantation methods. From left to right are the ASIA motor score, ASIA sensory score, and adverse reactions, respectively. [file 12967_2021_2843_MOESM1_ESM.doc]
